# Unraveling the Drivers of Tuberculosis: A Retrospective Panel Data Study Across 70 Developing Countries

**DOI:** 10.1002/hsr2.71192

**Published:** 2025-08-27

**Authors:** Md. Sazzad Hossain Mithu, Sodip Banik, Md Abir, Sk Shadi Joy, Md Jamal Uddin

**Affiliations:** ^1^ Department of Statistics Shahjalal University of Science & Technology Sylhet Bangladesh; ^2^ Faculty of Graduate Studies Daffodil International University, Savar Dhaka Bangladesh

**Keywords:** developing countries, global health, random‐effects model, risk factors, tuberculosis

## Abstract

**Background and Aims:**

Tuberculosis (TB) remains a major global cause of death, particularly in developing countries. This study aims to identify key risk factors contributing to high TB incidence in these nations, analyze regional variations, and assess how risk factors differ across continents.

**Methods:**

We conducted a retrospective analysis using data from 70 developing countries spanning 2000 to 2020, sourced from the World Bank Open Data. Variables included TB incidence, HIV prevalence, smoking rates, literacy rates, undernourishment, and population density. A random‐effects model was employed to examine the associations between these factors and TB incidence.

**Results:**

HIV prevalence (coefficient = 37.53, 95% CI: 34.28–40.79), smoking (3.51, 2.99–4.02), undernourishment (1.56, 1.02–2.10), and population density (0.16, 0.07–0.24) showed significant positive associations with TB incidence. Literacy rate was negatively associated with TB incidence (−0.11, −0.54 to 0.33), though not significantly. These findings highlight the strong influence of socio‐demographic and health‐related factors on TB burden.

**Conclusion:**

TB continues to pose a serious health challenge in developing countries. HIV control, reduction of undernourishment and smoking, and managing population density are critical to reducing TB incidence. Regional differences underscore the need for tailored prevention strategies.

## 1 Introduction

Tuberculosis (TB) is caused by the bacterium *Mycobacterium tuberculosis*. It primarily spreads through the air when a person with active pulmonary TB coughs, sneezes, speaks, or sings, releasing infectious droplets that can be inhaled by others. People with latent (inactive) TB infection do not spread the disease. Transmission risk is higher in enclosed spaces with poor ventilation [1, 2]. Pulmonary TB is one of the most harmful varieties of TB because of its effects on the lungs. After infecting the host, the bacilli rapidly multiply, threatening healthy tissues and weakening the immune system's ability to control the spread of the disease [3].

In 2016, TB caused an estimated 1.5 million deaths and 10.4 million new cases worldwide, according to the World Health Organization (WHO) [4]. By 2018, this burden persisted, with 10 million new cases reported globally and 1.5 million deaths [5]. In 2019, the WHO confirmed that TB remained the leading cause of death from a single infectious agent worldwide. Despite the emergence of the COVID‐19 pandemic in 2020, TB continued to pose a serious threat to global health [6]. The estimated number of global TB deaths between 2019 and 2021 showed a troubling increase due to the COVID‐19 pandemic, diverging from the previous downward trend observed from 2005 to 2019. In 2020, there was a significant global decline in newly confirmed TB cases compared to 2019, a decrease directly linked to the widespread impacts of the COVID‐19 pandemic. The total number of reported cases dropped by 18% from 2019 to 2020, reaching levels last seen in 2012, with 5.8 million cases registered in 2020 [7, 8].

In the HIV/AIDS community, TB is more than a health issue, it is a life‐threatening condition. It remains the leading cause of illness and death among people living with HIV, with 1.6 million recorded TB deaths in 2021. Of these, 187,000 were linked to HIV, while 1.4 million were unrelated. This increase marks a return to 2017 levels, following an estimated 1.4 million TB deaths in 2019 and 1.5 million in 2020 [3]. The majority of the 30 countries with the highest TB burden are developing nations, which account for most global TB cases and deaths. In these countries, newly diagnosed TB cases rose by 4.5%, from 10.1 million in 2020 to 10.6 million in 2021. Additionally, the TB incidence rate increased by 3.6% compared to the previous year, reversing the previous decline seen across most developing nations [3].

Numerous studies have demonstrated that various social, cultural, and health factors are linked to TB, contributing to its rising incidence. Among these factors, undernourishment significantly impacts TB incidence and mortality. As a major global risk factor, undernourishment is estimated to contribute to 2.2 million TB cases worldwide in 2022 [9, 10]. In addition, smoking, heavy alcohol consumption, and HIV prevalence appear to play a significant role in the TB incidence [11, 12]. WHO estimates that 1·3 billion people smoke worldwide, with severe regulations and public health initiatives. The majority of these smokers reside in nations like China, India, Bangladesh, Vietnam, the Philippines, and the Russian Federation because TB is a prevalent illness there [13]. Smoking is associated with a ninefold increase in the chance of dying from TB, but quitting can reduce this risk by up to 65% [14].

There is currently no research on how TB risk factors vary geographically. Understanding whether these key factors differ across continents is a central focus of our study. The primary objective of this study was to assess the impact of risk factors contributing to the high incidence of TB in developing nations and to examine regional patterns. Additionally, this study aimed to determine how these risk factors vary across continents. Our findings provide valuable insights to support policymakers and healthcare practitioners in developing nations, helping them take more effective measures to address this critical issue.

## 2 Materials and Methods

### 2.1 Data Source

The study, conducted on a global scale, focused on the unique health challenges faced by developing countries. Information on the TB incidence, HIV Prevalence, smoking, literacy rate, undernourishment, and population density in these nations from 2000 to 2020 was meticulously gathered from the World Bank Database (World Bank Open Data | Data) and the period between 2000 and 2020 witnessed significant advancements in TB research, leading to the creation of novel diagnostic instruments, medications, and vaccinations.

Developed countries were intentionally excluded from this study to underscore the unique health challenges faced by developing nations. These countries often grapple with inadequate health infrastructure, limited access to health care services, and a high burden of communicable diseases like TB. This is in stark contrast to developed countries, which do not face these disparities. The disproportionate impact of global health challenges such as TB on developing countries, with higher incidence and mortality rates when compared to developed countries, underscores the urgency and importance of researching these nations. Such research can provide crucial insights into the efficacy of public health policies and interventions in resource‐constrained environments.

### 2.2 Data Harmonization and Sample Size

The final sample included 70 developing countries like Afghanistan, Albania, Algeria, Argentina, Azerbaijan, Bangladesh, Belarus, Belize, Benin, Bolivia, Botswana, Brazil, Bulgaria, Burkina Faso, Cambodia, Cameroon, Chad, Colombia, Costa Rica, Cote d'Ivoire, Cuba, Dominican Republic, Ecuador, El Salvador, Ethiopia, Fiji, Georgia, Ghana, Guatemala, Guyana, India, Indonesia, Jamaica, Kazakhstan, Kenya, Kyrgyz Republic, Lebanon, Liberia, Madagascar, Malawi, Malaysia, Mali, Mauritania, Mauritius, Mexico, Montenegro, Morocco, Myanmar, Nepal, Nigeria, Oman, Pakistan, Paraguay, Papua New Guinea, Peru, Philippines, Romania, Rwanda, Sao Tome and Principe, Senegal, Serbia, Sierra Leone, South Africa, Sri Lanka, Tanzania, Thailand, Timor‐Leste, Togo, Tunisia, Ukraine. The classification of countries as “developing” was based on the World Bank's income classification, which includes low, low‐middle, and upper‐middle income economies [15]. Since the focus of the research is TB, it is essential to concentrate on countries where TB remains a significant public health concern. This study gathered data for each variable from the World Bank's database from 2000 to 2020 and conducted a country‐level data aggregation process. The World Bank obtains primary information from international organizations, national statistical organizations, central banks, customs services, and other government agencies that create the statistics in the World Development Indicators database.

### 2.3 Study Variables

All variables included in this study were continuous variables (Table 1).

#### 2.3.1 Outcome

As an outcome variable, we used the TB incidence rate, which refers to the estimated number of new and relapse TB cases arising in a given year, expressed as the rate per 100,000 population (Incidence of tuberculosis (per 100,000 people) | Data).

**Table 1:** Overview of the variables.

Variables	Measurement
TB incidence rate	TB incidence (per 100,000 people)
HIV	Prevalence of HIV, ages 15–49 (per 1000 uninfected population)
Smoking	Prevalence of current tobacco use (% of adults)
Undernourishment	Prevalence of undernourishment (% of population)
Literacy rate	Literacy rate, adult total (% of people ages 15 and above)
Population density	Population density (people per sq. km of land area)

#### 2.3.2 Explanatory Variables

HIV, undernourishment, smoking, population density, and literacy rate are the explanatory variables. HIV measures the percentage of individuals aged 15–49 with HIV infection for every 1000 individuals in the uninfected population within the same age group. Undernourishment, as defined by the World Bank, refers to the percentage of the population whose habitual food consumption is insufficient to provide the dietary energy levels that are required to maintain a normal, active, and healthy life (Prevalence of undernourishment (% of population) | Data).

Smoking refers to the prevalence of current tobacco use among adults, expressed as the percentage of the population aged 15 years and over who currently use any tobacco product (smoked and/or smokeless) on a daily or non‐daily basis. Tobacco products include cigarettes, cigars, pipes, waterpipes (hookah, shisha), bidis, kretek, heated tobacco products, and all forms of smokeless (oral and nasal) tobacco. This measure excludes e‐cigarettes and related non‐tobacco electronic devices. The data were obtained from the World Bank Open Data portal, originally sourced from the WHO Global Health Observatory. These estimates are derived using a Bayesian negative binomial meta‐regression model and are age‐standardized to the WHO Standard Population to ensure international comparability. No primary data collection was conducted by the authors; only secondary data were used (Prevalence of current tobacco use (% of adults) | Data).

Literacy rate measures the percentage of the population aged 15 years and above who can both read and write, with understanding, a short and simple statement about their everyday life. It is considered an important indicator of educational attainment and is used as a proxy for human capital and socioeconomic development. The data were obtained from the World Bank Open Data portal, sourced from the UNESCO Institute for Statistics. Estimates are based on national censuses and household surveys, with some adjustments using the Global Age‐Specific Literacy Projection Model (GALP) for countries lacking recent data. This variable is relevant in TB research as literacy may influence awareness of health risks, health‐seeking behaviors, and the ability to access healthcare services (Literacy rate, adult total (% of people ages 15 and above) | Data).

Population Density measures the number of people living per square kilometer of land area in a country. It is calculated by dividing the midyear population by the total land area (excluding inland water bodies such as rivers and lakes). The population figures follow the *de facto* definition, meaning they include all residents regardless of legal status or citizenship, excluding refugees not permanently settled in the country of asylum. The data were obtained from the World Bank Open Data platform, originally compiled from the Food and Agriculture Organization and World Bank population estimates. In the context of this study, population density is used as a proxy for crowding and urban pressure, which may facilitate the transmission of infectious diseases like TB (Population density (people per sq. km of land area) | Data).

Time is considered a numerical predictor variable.

### 2.4 Data Analyses

In this study, we used a panel data approach with secondary data. Panel data combines cross‐sectional and time‐series information, offering a longitudinal structure that captures variability across both time and countries [16]. We constructed the data set by merging data based on year and country identifiers.

We investigated the association between the independent variables (literacy rate, HIV prevalence, smoking, undernourishment, and population density) and the outcome variable (TB incidence rate) using a random‐effects model. This model accounts for unobserved heterogeneity across countries by treating the country as a random effect, enabling generalization beyond the specific countries analyzed.

To minimize the potential confounding effects among the explanatory variables, we used a multivariable random‐effects model, which allows for simultaneous adjustment and estimates the independent effect of each predictor on TB incidence. We assessed multicollinearity using correlation matrices and variance inflation factors (VIFs), all of which were below the commonly accepted threshold of 5, confirming acceptable independence among predictors (Table 2).

**Table 2:** Multicollinearity table.

Variables	Variance inflation factor (VIF)
HIV	1.109
Undernutrition	1.481
Smoking	1.593
Population density	1.214
Literacy rate	1.284

We first performed univariable analyses to explore the bivariate relationships between each explanatory variable and TB incidence. However, for the final multivariable model, we included all pre‐specified variables, irrespective of their statistical significance in the univariable analysis, based on theoretical relevance and prior literature.

To ensure data quality, we handled missing values using forward and backward imputation techniques. Outliers were identified and removed using the *Z*‐score method, where any data point more than three standard deviations from the mean was excluded.

All statistical tests were two‐sided, with a significance level of *α* = 0.05. *p*‐values < 0.001 were reported as “*p* < 0.001,” values between 0.001 and 0.01 were reported to three decimal places, and values ≥ 0.01 were reported to two decimal places. We emphasized effect sizes and 95% confidence intervals (CIs) over *p*‐values alone to present a more meaningful interpretation of the results.

All data processing and statistical analyses were conducted using a combination of Python and R. Data preparation and outlier detection were carried out in Python (v3.11.9) using the libraries Pandas (v1.5.1) and NumPy (v1.23.4). All statistical analyses and modeling were performed in R (v4.4.2) utilizing the following packages: plm (v2.6.6) for panel data analysis, Kendall (v2.2.1) for non‐parametric correlation testing, and car (v3.1.3) for diagnostic tests, including multicollinearity assessment. All figures were generated using Plotly.js (v3.0.1) except Figure 5, which was created by Excel.

We followed the SAMPL (Statistical Analyses and Methods in the Published Literature) guidelines to ensure transparent and reproducible reporting of statistical methods and results.

This study adhered to the STROBE (Strengthening the Reporting of Observational Studies in Epidemiology) guidelines to promote transparency, replicability, and completeness in reporting. Ethical approval and informed consent were not required, as no direct involvement of human participants was present.

### 2.5 Pre‐Specified and Exploratory Analyses

The random effects model examining the relationship between the selected independent variables and TB incidence was pre‐specified based on existing literature and hypotheses. No exploratory or subgroup analyses were performed.

## 3 Result

This section will initially cover descriptive statistics about the variables. The incidence rate of TB was the dependent variable. In this case, the independent variables include population density, smoking, HIV, undernourishment and literacy rate.

The analysis of 70 nations revealed a wide variety of incidence rates for TB, with a median of 99 cases per 100,000 individuals and an interquartile range (IQR) of 192.75. This large IQR demonstrates the wide variation in TB incidence rates throughout countries, with rates varying dramatically. The prevalence rate of HIV also varies significantly between countries, with a median of 0.4 cases per 1,000 uninfected people and an IQR of 1.3, indicating there are significant variations in HIV prevalence, with some nations having rates that are significantly higher or lower than the median. The rates of undernourishment exhibit a significant range as well, with an IQR of 13.275 and a median of 8.8%. This wide IQR shows considerable differences in undernourishment rates among the nations, with some having much higher levels of food insecurity than others. The smoking rate, with a median of 23.1% and an IQR of 18.8, exhibits a similar pattern of variance. Even though the variance is slight, it nevertheless indicates significant variations in the prevalence of smoking among the various nations. With a median of 72.2 persons per square kilometer and an IQR of 91.432, population density likewise ranges, showing how unevenly distributed the population is among the nations. In conclusion, there is a great deal of variation in literacy rates, with a median of 85.0% and an IQR of 33.556. This wide IQR indicates that literacy rates vary significantly between countries, with some attaining universal literacy and others having far lower rates. Table 3 shows the descriptive results.

**Table 3:** Descriptive statistics table.

Variables	First quartile (Q1)	Median (Q2)	Third quartile (Q3)	IQR = Q3 − Q1	Maximum	Minimum
TB incidence rate	40.250	99.000	233.00	192.750	914.00	3.100
HIV	0.100	0.400	1.400	1.300	25.900	0.100
Undernourishment	5.000	8.800	18.275	13.275	45.600	2.500
Smoking	13.400	23.100	32.200	18.800	66.900	3.500
Literacy rate	61.310	84.994	94.866	33.556	99.974	21.823
Population density	36.289	72.218	127.721	91.432	1265.187	2.552

In 2000, Botswana had the highest TB incidence rate, with 914.000 cases, while Jamaica had the lowest TB incidence rate, with 3.100 instances in 2020. The highest recorded HIV prevalence rate was 25.900 in 2001 in Botswana, while the lowest rate was 0.100 per 1000 uninfected people in several other countries. Undernourishment reached its highest point in Afghanistan in 2002 at 45.600%, while the lowest recorded figure for several countries during the same year was 2.500%. With 66.900% of people reporting using tobacco in 2004, Myanmar had the highest prevalence, while Ghana had the lowest, with 3.500% reported in 2020. Bangladesh is the most densely populated nation, with over 1265.187 inhabitants per square kilometer of land, whereas Mauritania is home to only approximately 3 people. Burkina Faso had the lowest literacy rate (21.823%), and Ukraine had the highest (99.974%).

We conducted a random effect model to assess the effect of independent variables (HIV, smoking, undernourishment, literacy rate, and population density) on the dependent variable (TB incidence rate).

From Table 4, we can see that HIV prevalence was positively associated with TB incidence (estimate: 37.54, 95% CI: 34.28–40.79, *p* < 0.001). This indicates that a one‐unit increase in HIV prevalence corresponds to an increase of approximately 37 TB cases per 100,000 population, controlling for other variables. Undernourishment was also positively associated with TB incidence (estimate: 1.56, 95% CI: 1.02–2.10, *p* < 0.001), suggesting that a 1% increase in undernourishment prevalence is associated with about 1.56 additional TB cases per 100,000 people. Smoking prevalence showed a positive association with TB incidence (estimate: 3.51, 95% CI: 2.99–4.02, *p* < 0.001), meaning that a 1% increase in smoking prevalence is estimated to increase TB incidence by approximately 3.5 cases per 100,000 population. Population density was positively associated with TB incidence (estimate: 0.16, 95% CI: 0.07–0.24, *p* < 0.001), suggesting that for each unit increase in population density, TB incidence rises by about 0.16 cases per 100,000 population, adjusting for other factors.

**Table 4:** Estimation outcome of random effect model.

	Estimate	95% CI	Standard error	*Z*‐value	*p* value
(Intercept)	−10.711	[−56.883, 35.461]	23.577	−0.455	0.65
HIV	37.533	[34.283, 40.787]	1.659	22.625	*p* < 0.001
Undernourishment	1.559	[1.018, 2.100]	0.276	5.649	*p* < 0.001
Smoking	3.510	[2.994, 4.024]	0.263	13.364	*p* < 0.001
Population density	0.157	[0.073, 0.240]	0.042	3.687	*p* < 0.001
Literacy rate	−0.106	[−0.540, 0.327]	0.221	−0.481	0.63

Literacy rate showed a negative but nonsignificant association with TB incidence (Estimate: −0.11, 95% CI: −0.54 to 0.33, *p* = 0.34), indicating no clear evidence that literacy rate influences TB incidence in this model.

The model explained approximately 46.1% of the variance in TB incidence (*R*² = 0.461, adjusted *R*² = 0.459), indicating a moderate fit to the data.

The trend of the TB incidence rate in Asia, Africa, Europe, South America, and Oceania is significant except for North America (Table 5). North and South America have lower TB incidence rates than other locations. Oceania has the highest TB incidence rate, and North America has the lowest.

**Table 5:** Output of Mann–Kendall non‐parametric test.

Region	tau	*p* value
Asia	−1	*p* < 0.001
Africa	−0.848	*p* < 0.001
Europe	−0.943	*p* < 0.001
North America	−0.00957	0.976
South America	−0.711	*p* < 0.001
Oceania	0.848	*p* < 0.001
World	−0.981	*p* < 0.001

**Figure 1:**
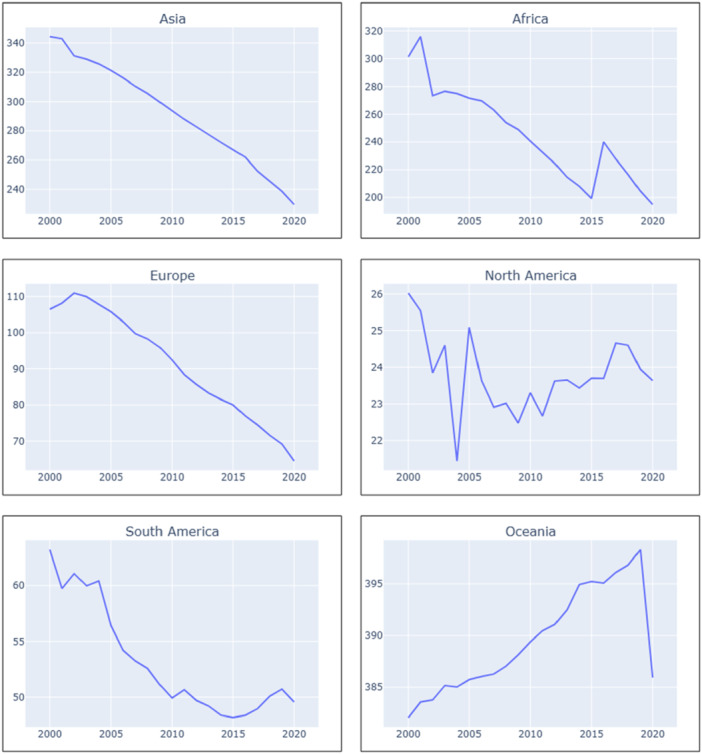
Trends of TB incidence rates across diverse geographic regions (2000–2020) show the trend of TB incidence rates across diverse geographic regions. The *X*‐axis represents the time considered in this study (2000–2020), and the *Y*‐axis represents the incidence rates of TB per 100,000 people. *Source:* Author's analysis using data from the World Bank.

The TB incidence rate in the Asian region gradually declined throughout the period. The African region experienced an increasing trend from 2000 to 2001 and 2015 to 2016, with a decreasing trend observed in the rest of the years. North America showed a fluctuating trend, with periodic increases and decreases in TB incidence rate. We initially observed an increasing trend in Europe and South America, but both regions made noteworthy progress. Region Oceania consistently maintained a high TB incidence rate throughout the entire period (Figure 1).

## 4 Discussion

**Figure 2:**
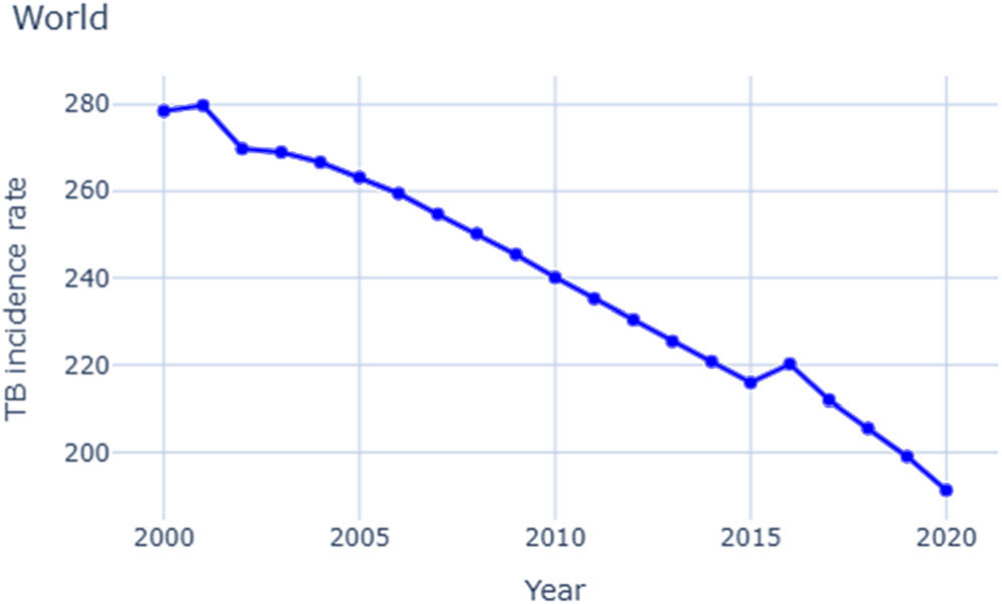
World tuberculosis prevalence rate trends (2000–2020) show the trend of world TB prevalence rate from 2000 to 2020. *Source:* Author's analysis using data from the World Bank.

In this study, we explore common factors associated with TB across different regions and analyze the trend of the TB incidence rate from 2000 to 2020. This study is a crucial tool in understanding how TB incidence has evolved over the past two decades, providing valuable insights and knowledge. Our findings revealed that TB is still a burden for many developing countries. HIV prevalence, smoking, undernourishment, and population density are the risk factors influencing TB incidence, according to a similar statement given in another study [11]. From 2000 to 2020, we observed a gradual decline in TB incidence rates per 1,000,000 people globally (Figure 2). This decline suggests that global efforts to control and prevent TB progress are being made, but it also underscores the need for continued vigilance and investment in TB control programs to sustain this positive trend.

**Figure 3:**
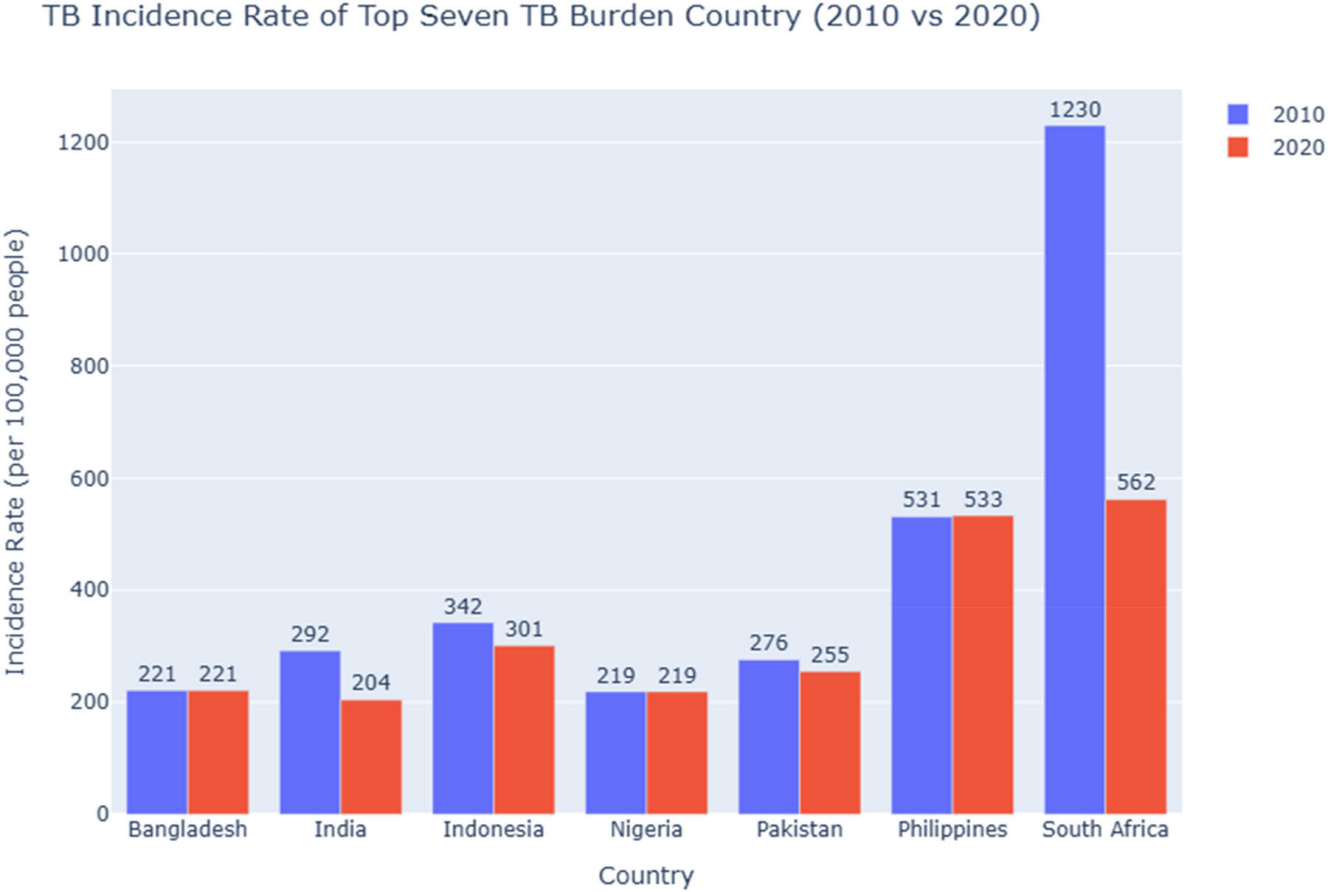
Comparison of TB incidence rate among the top seven TB burden countries (2010 vs. 2020) compares TB incidence rates of the seven TB burden countries between 2010 and 2020. The *X*‐axis represents the country name, and the *Y*‐axis represents the incidence rate of TB per 100,000 people. *Source:* Author's data analysis using data from the World Bank.

Between 2010 and 2020, significant reductions in TB incidence were observed in India (292 to 204), Indonesia (342 to 301), Pakistan (276 to 255), and South Africa (1230 to 562), reflecting the impact of strengthened TB control measures in these countries. In contrast, the Philippines experienced a slight increase (531 to 533), while Bangladesh and Nigeria showed no change, maintaining rates of 221 and 219 per 100,000 people, respectively. Despite retaining the highest incidence rate in 2020, South Africa exhibited the most substantial absolute decline—over 50%—highlighting notable progress (Figure 3). These differences reflect the varied effectiveness of national TB control strategies among high‐burden countries.

**Figure 4:**
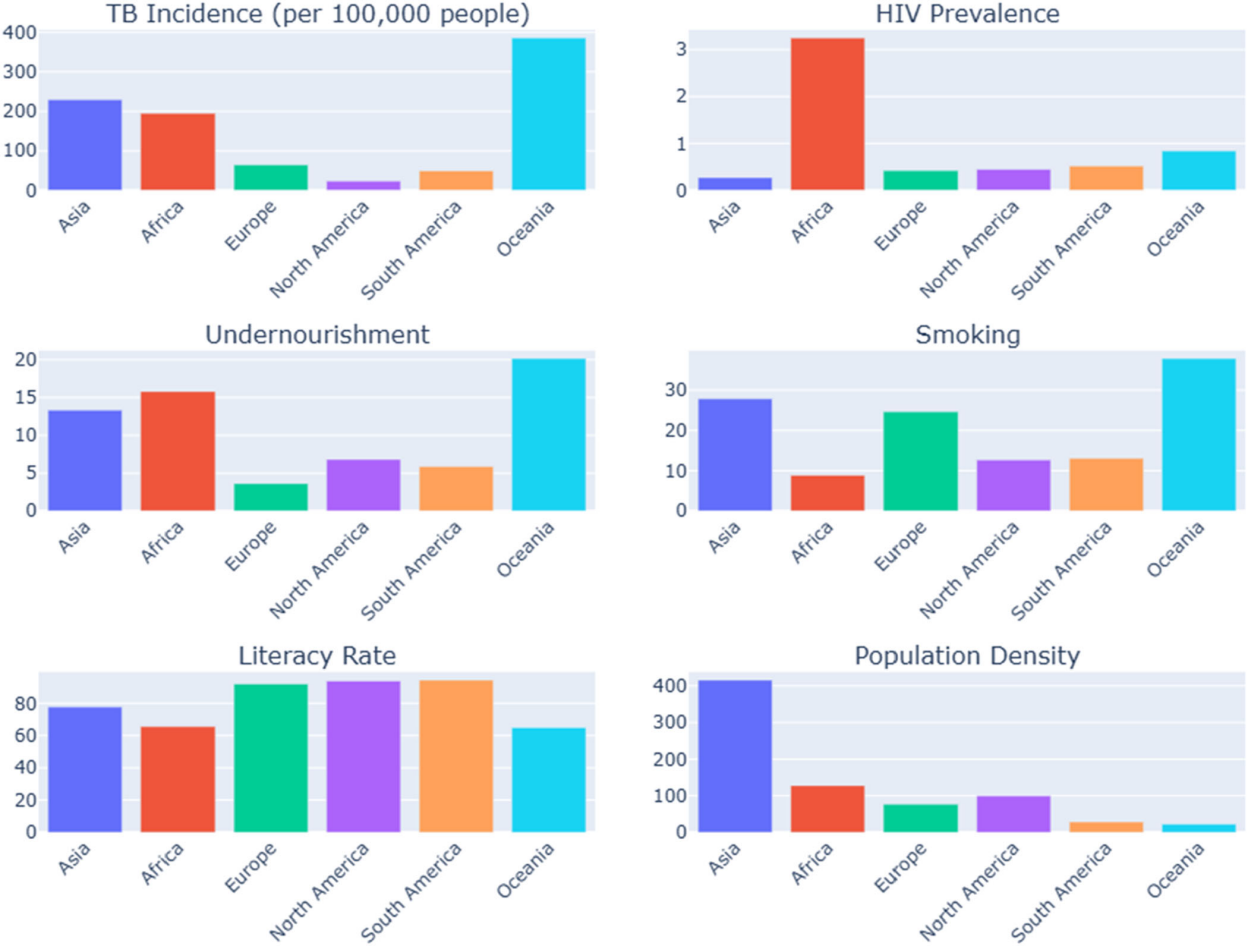
Regional comparison of study variables in 2020 shows a bar plot for a region‐wise comparison of each study variable in 2020. Each bar represents a particular region. *X*‐axis represent regions, and the *Y*‐axis represents measures of study variables. *Source:* Author's analysis using data from the World Bank.

Our data indicate a substantial correlation between TB incidence and HIV prevalence, suggesting that the risk of TB is higher in individuals living with HIV than in those not living with HIV [17]. According to our study, several African countries, including Botswana, Malawi, Kenya, South Africa, Cameroon, and Cote d'Ivoire, exhibit a notably higher prevalence of HIV and TB incidence rates (Figure 4). Countries with low HIV prevalence and TB incidence rates include Tunisia, Oman, Montenegro, Lebanon, Bulgaria, Serbia, Sri Lanka, Albania, Algeria, Fiji, and others. National HIV prevalence levels in South Asia are relatively low compared to other regions, particularly Africa.

Apart from Asia, each continent has shown an upward trend, as per our data. Several nations with low HIV prevalence (Afghanistan, Bangladesh, Pakistan, Nepal, Indonesia, India, Myanmar, Timor‐Leste, the Philippines, and Madagascar) have high TB incidence rates. Notably, these nations have implemented effective HIV prevention programs, which have shown promising results in reducing the spread of the virus among high‐risk groups such as sex workers, injecting drug users, men who have sex with men, and transgender women. These programs have played a critical role in limiting the spread of HIV in these settings [18].

Our findings revealed that undernourishment is the leading risk factor for TB and hurts TB incidence rates. Those with a healthy body weight are less likely to develop active TB than those who are undernourished. Previous research reported similar findings [19, 20, 21, 22]. Among the countries in our study, Liberia, Rwanda, Chad, Timor‐Leste, Tanzania, Kenya, and Papua New Guinea have averaged high undernourishment over the past two decades. On the other hand, countries like South Africa, Romania, Cuba, Ukraine, Belarus, Serbia, Malaysia, Argentina, and Tunisia have averaged low undernourishment. Evidence suggests that countries with high undernourishment have higher TB incidence rates compared to countries with lower undernourishment. According to our data, Asian, African, and Oceanian countries have higher undernourishment than European, North American, and South American countries (Figure 4). Among them, Oceania has the highest prevalence, and Europe has the lowest prevalence of undernourishment. The prevalence of undernourishment shows a downward trajectory in all regions. Overcoming the prevalence of undernourishment can aid in the reduction of TB incidence. Action by the government is required in six primary areas to address the nutrition crisis, such as establishing resilient, sustainable food systems for healthy diets; giving social security and nutrition education to all; aligning health systems with nutrition needs; delivering universal coverage for crucial nutrition interventions, such as vitamin supplementation and fortified foods; ensuring that trade and investment policies promote nutrition; creating safe and supportive settings for nutrition at all ages; and improving and promoting nutrition governance and accountability globally (*WHO*).

Consistent with the previous research, a noteworthy positive correlation was found between smoking and TB incidence rates [14, 23]. Countries with higher TB incidence rates have a higher prevalence of smoking (Philippines, Botswana, Timor‐Leste, Myanmar, Cambodia, Papua New Guinea, Indonesia, India, Pakistan, and Nepal, among others). We also observed that Asian countries have a higher prevalence of smoking than other regional countries, which significantly affects TB incidence rates compared to other risk factors (Figure 4). Countries with high TB burdens, like India, Pakistan, Bangladesh, Indonesia, and the Philippines, have high smoking rates. Few countries with higher smoking rates have lower TB incidence rates (Cuba et al.). The results mentioned earlier could be the result of several factors. First, nations with high smoking rates have put in place comprehensive and successful TB control strategies. These initiatives include early detection, treatment accessibility, contact tracing, and infection control methods that can effectively stop the spread of TB, even in areas where smoking is highly prevalent [24]. Second, countries with low TB incidence rates may have robust immunization programs, notably for childhood immunization against TB utilizing the Bacillus Calmette‐Guérin (BCG) vaccine. In some areas, BCG vaccination may help reduce TB incidence rates by offering some degree of protection against TB infection [25, 26, 27]. Effective public health measures, such as smoking cessation and TB prevention programs, can help reduce the influence of smoking on TB incidence. These treatments can target high‐risk populations and promote healthy behaviors [14]. Access to quality healthcare and early detection and treatment of TB can help prevent the disease from progressing to an active disease [28]. Developed healthcare systems may better position countries to manage and control TB cases. Countries with high smoking rates but low TB incidence may have better overall living conditions, less overcrowding, and greater access to healthcare, all of which can minimize the risk of TB transmission.

Our study found that areas with higher population densities tend to have higher TB incidence cases, a trend that is consistent with previous research [29]. This finding has significant global implications, as it suggests that countries with high population densities, such as Bangladesh, India, Pakistan, and the Philippines, are more likely to experience high TB incidence rates. Conversely, countries with lower population densities, such as Guyana, Oman, Mali, Belize, Fiji, Azerbaijan, Malaysia, Algeria, Benin, Burkina Faso, Tunisia, Belarus, Albania, Mexico, Argentina, and Brazil, tend to report lower TB incidence rates. Despite the general trend of higher population densities in Asian countries, there are exceptions. For example, Cambodia, Myanmar, Timor‐Leste, Botswana, Kenya, and Tanzania have higher TB incidence rates despite their low population densities. This variability is particularly pronounced in the Asian and African regions.

This study found a negative correlation between TB incidence and literacy rates. Other research supports these findings [30, 31]. Countries like Jamaica, Cuba, Oman, Lebanon, Mauritius, Costa Rica, Albania, Mexico, Montenegro, and Guatemala, with high literacy rates, tend to have low TB incidence rates. In contrast, nations like Afghanistan, Chad, Ethiopia, Liberia, South Africa, and Papua New Guinea, with lower literacy rates, tend to have high TB incidence rates. Some countries, including Cambodia, India, Indonesia, Myanmar, the Philippines, Botswana, Kenya, and Tanzania, have high literacy and TB incidence rates. A pronounced inverse relationship between literacy and TB incidence rates was observed in North and South America (Figure 4). Despite this general trend, variability exists, particularly in Asian and African countries. Several factors explain these discrepancies. First, in impoverished nations where poverty and limited healthcare access are primary TB drivers, the influence of literacy might be less emphasized [32]. Second, the healthcare quality and reach in underdeveloped countries vary, with many TB cases potentially going undiagnosed or untreated, irrespective of the community's literacy levels, thereby muting literacy's impact on TB incidence [33]. Government policies and health initiatives also mitigate the influence of literacy on TB incidence rates. Due to these interventions, countries with robust TB control programs might see a reduced effect on literacy. Lastly, considering TB's lengthy incubation period, shifts in literacy might not immediately reflect in TB trends. Therefore, long‐term patterns and potential lag effects should be acknowledged when exploring the link between literacy and TB incidence [34].

**Figure 5:**
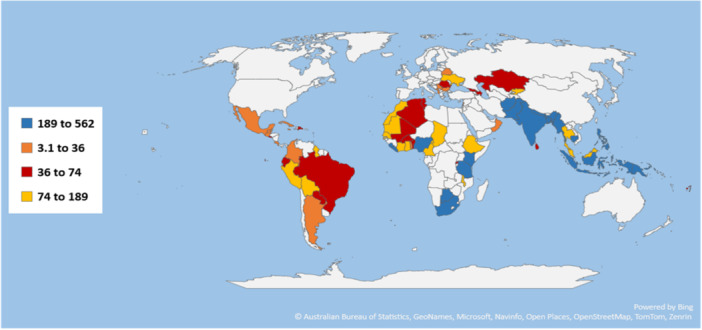
Distribution of tuberculosis in the world in 2020. Countries with white colors are not included in the study, and countries with different color shades represent corresponding categories of TB incidence rate. *Source:* Author's data analysis using data from the World Bank.

In the existing literature, TB incidence rates have been studied by looking at individual or group‐specific factors, typically within specific countries or continents. In contrast, our study takes a comprehensive approach, considering a range of factors such as socio‐economic determinants, healthcare influences, and the interactions among these factors across different continents (Figure 5). Furthermore, our research goes beyond a single snapshot, conducting a longitudinal analysis of TB incidence rates from 2000 to 2020 across various continents. This novel research paradigm fills a significant void in the current TB research landscape. Our study uncovers previously unnoticed regional variations and trends. For instance, we have observed a marked decline in TB incidence rates within the Asian region, which contrasts sharply with the upward trajectory shown on the Oceanian continent. Also, we have identified noteworthy disparities in the behavior of factors among different continents and even within countries sharing the same geographical space. These regional inequalities call into question the conventional view of TB epidemiology, underscoring the importance of personalized, region‐specific therapies. Our findings have far‐reaching consequences. We provide essential insights for policymakers and healthcare practitioners through our rigorous investigation of the varied nature of TB incidence rates and the dissection of regional trends. Our findings highlight the critical need to customize TB prevention and control measures to the specific characteristics of each location and its population. It emphasizes the urgent need for increased investment in hospital facilities and socio‐economic development, particularly in areas with the highest TB burden. TB rates differ from place to place, which makes it crucial to understand this disease better. Our research encourages action, pushing everyone to take a step to combat TB. It is essential to approach TB differently in each region based on its unique needs, and we urge everyone to play their part in this crucial fight.

## 5 Limitations of the Study

Although this study offers insightful information about the variables impacting TB prevalence rates in underdeveloped nations, it is crucial to recognize several limitations that could affect how the results are interpreted and applied in broader contexts. The data used, primarily sourced from the World Bank, may contain reporting inconsistencies and inaccuracies, potentially impacting the accuracy of the results. The exclusion of certain developing countries due to missing data and the omission of developed countries may introduce selection bias, limiting the broader applicability of the findings. The imputation of missing data using forward and backward methods assumes specific patterns of missingness and may not fully account for the complexity of the missing data. The study's focus on a limited set of variables omits potentially relevant factors, such as healthcare infrastructure and cultural determinants, which may influence TB incidence rates. The study's period from 2000 to 2020 may not capture recent developments or shifts in TB dynamics and related factors, affecting the study's current relevance. The small sample size of 70 developing countries may limit the ability to detect significant relationships between variables. Also, the study may have an ecological bias. Environmental factors such as temperature and humidity were not included in our analysis. Future studies could investigate their influence on TB transmission, as climate conditions may affect bacterial survival and population behavior.

## 6 Conclusion

Despite progress, TB continues to be a significant burden in many developing countries. Risk factors for TB include HIV, undernourishment, smoking, and population density. From 2000 to 2020, global TB rates decreased, with the lowest rates observed in North and South America and the highest in Oceania. Individuals living with HIV are at a significantly increased risk of contracting TB; hence, controlling HIV prevalence could help reduce TB incidence. African countries report high rates of both HIV and TB. Countries with widespread undernourishment also tend to report higher TB incidence rates, with Asian, African, and Oceanian countries having greater undernourishment compared to Europe, North America, and South America. Addressing undernourishment could play a vital role in reducing TB incidence. Smoking is another contributing factor, with Asian countries displaying higher smoking rates, which might lead to increased TB incidence. However, certain countries with high smoking rates, such as Cuba and Lebanon, show lower TB incidence rates, attributed to effective TB control measures. While Asian countries tend to have higher population densities, some Asian and African countries present anomalies with increased TB rates despite lower population densities, underscoring the intricate interplay between population density and TB incidence. Our study discerned a negative correlation between literacy rates and TB incidence. This negative link is noticeable in North America, South America, and Oceania, while there are anomalies in the Asian and African regions. Managing population growth, improving nutritional education, and reducing tobacco use are some strategies to lessen the incidence of TB. The study emphasizes that socio‐demographic factors, such as Population density, are crucial in predicting the TB incidence, in addition to medical determinants like smoking and HIV.

## Author Contributions

**Md Sazzad Hossain Mithu:** conceptualization, investigation, writing – original draft, methodology, validation, visualization, software, formal analysis, data curation, supervision. **Sodip Banik:** writing – original draft, conceptualization, methodology, investigation, validation. **Md Abir:** investigation, visualization, formal analysis, conceptualization, data curation, validation. **Sk Shadi Joy:** investigation, writing – original draft, validation. **Md Jamal Uddin:** validation, writing – review and editing, project administration, supervision. All authors have read and approved the final version of the manuscript.

## Conflicts of Interest

The authors declare no conflicts of interest.

## Transparency Statement

The lead author, Md Jamal Uddin, affirms that this manuscript is an honest, accurate, and transparent account of the study being reported; that no important aspects of the study have been omitted; and that any discrepancies from the study as planned (and, if relevant, registered) have been explained.

## Data Availability

The data that support the findings of this study are available from the corresponding author upon reasonable request.
